# Exploitation of Natural By-Products for the Promotion of Healthy Outcomes in Humans: Special Focus on Antioxidant and Anti-Inflammatory Mechanisms and Modulation of the Gut Microbiota

**DOI:** 10.3390/antiox13070796

**Published:** 2024-06-29

**Authors:** Luigi Santacroce, Lucrezia Bottalico, Ioannis Alexandros Charitos, Francesca Castellaneta, Elona Gaxhja, Skender Topi, Raffaele Palmirotta, Emilio Jirillo

**Affiliations:** 1 Section of Microbiology and Virology, Interdisciplinary Department of Medicine, School of Medicine, University of Bari Aldo Moro, 70124 Bari, Italy; raffaele.palmirotta@uniba.it (R.P.); emilio.jirillo@uniba.it (E.J.); 2 Department of Clinical Disciplines, University ‘Alexander Xhuvani’ of Elbasan, 3001 Elbasan, Albaniaelona.gaxhja@uniel.edu.al (E.G.); skender.topi@uniel.edu.al (S.T.); 3 Istituti Clinici Scientifici Maugeri IRCCS, Pneumology and Respiratory Rehabilitation Unit, Institute of Bari, 70124 Bari, Italy; alexanestesia@hotmail.com; 4 School of Clinical Biochemistry and Pathology, University of Bari Aldo Moro, 70124 Bari, Italy

**Keywords:** gut microbiota, food by-products, antioxidant activity, anti-inflammatory activity, healthcare, circular economy

## Abstract

Daily, a lot of food is wasted, and vegetables, fruit, and cereals as well as marine products represent the major sources of unwanted by-products. The sustainability, waste recovery, and revalorization of food by-products have been proposed as the main goals of the so-called circular economy. In fact, food wastes are enriched in by-products endowed with beneficial effects on human health. Grape, olives, vegetables, and rice contain different compounds, such as polyphenols, dietary fibers, polysaccharides, vitamins, and proteins, which exert antioxidant and anti-inflammatory activities, inhibiting pro-oxidant genes and the Nuclear Factor kappa-light-chain-enhancer of activated B cells (NF-kβ) pathway, as demonstrated by in vitro and in vivo experiments. Dietary fibers act upon the gut microbiota, expanding beneficial bacteria, which contribute to healthy outcomes. Furthermore, marine foods, even including microalgae, arthropods, and wastes of fish, are rich in carotenoids, polyphenols, polyunsaturated fatty acids, proteins, and chitooligosaccharides, which afford antioxidant and anti-inflammatory protection. The present review will cover the major by-products derived from food wastes, describing the mechanisms of action involved in the antioxidant and anti-inflammatory activities, as well as the modulation of the gut microbiota. The effects of some by-products have also been explored in clinical trials, while others, such as marine by-products, need more investigation for their full exploitation as bioactive compounds in humans.

## 1. Introduction

There is growing evidence that a large amount of industrial food is wasted as unwanted by-products, with their enormous accumulation in the environment [1]. For example, the waste of fruit and vegetables is equal to 50% of production in the processing and post-harvest period [2]. To overcome the above-cited problem, the circular economy has been proposed as an effective strategy to promote the sustainability, waste recovery, and revalorization of by-products [3,4]. In fact, many food wastes are enriched with chemical compounds endowed with nutritional and bioactive functions, as in the case of plant- and marine-derived by-products [5,6]. The above-indicated by-products are enriched in phenols, carotenoids, phytosterols, polysaccharides, proteins, fatty acids, vitamins, and minerals, which may be beneficial in the management of inflammatory and degenerative diseases [7]. Then, modern industry is involved in the recycling and reuse of food by-products, which is based on the replacement of synthetic products with natural extracts for preventive and therapeutic use [8]. Of note, the effective recovery of bioactive products depends on certain variables, such as type of cultivar, type of culture methods, weather, sunlight, and exposure of plants to infectious agents, insects, and pesticides.

With special reference to the properties exerted by food by-products, antioxidant and anti-inflammatory activities are the most predominant. Thus, their administration may be beneficial in the case of oxidative and inflammatory conditions. Oxidative stress with the generation of reactive oxygen species (ROS) leads to harmful effects in the host, such as cellular membrane damage, alteration in the intestinal permeability, and endotoxemia [9]. Moreover, chronic exposure to stress due to lifestyle, cigarette smoking, and radiation may lead to aging and neurodegenerative disorders through the release of oxidants [10].

Oxidative stress and inflammation are intertwined with each other, as in the case of intestinal inflammation [11]. For instance, mucosal infiltration with neutrophils gives rise to ROS generation, migration of immune cells to the epithelium, and production of inducible nitric oxide synthase (iNOS) [12]. In addition, ROS act upon Toll-Like Receptors (TLRs), thus permitting the translocation of Nuclear Factor kappa-light-chain-enhancer of activated B cells (NF-kβ) to the nucleus, with the release of pro-inflammatory cytokines, adhesion molecules, and inflammatory enzymes [13]. Among pro-inflammatory mediators, interleukin (IL)- 1β, IL-6, IL-8, and Tumor Necrosis Factor (TNF)-α are the most prevalent during inflammation (e.g., inflammatory bowel disease and rheumatoid arthritis), thus representing appropriate targets for natural products [14].

A comprehensive analysis of the current literature, through searching the related biological and clinical data on major scientific databanks, e.g., Scopus, Clarivate Analytics, PubMed, and EMBASE, also using the ‘cited by’ and ‘similar articles’ options available in PubMed, was carried out to prepare this review. All relevant data have been selected and reported after a critical appraisal.

In the present review, emphasis will be placed on the beneficial effects of certain by-products from fruit, vegetables, cereals, and marine products with special reference to their antioxidant and anti-inflammatory activities, and modulation of the gut microbiota.

## 2. Major By-Products Extracted from Fruit, Vegetables, and Cereals

Daily, tons of vegetables, fruits, and cereals are discarded as undesired foods, as well as during industrial processing to make oil, wine, pasta, bread, and juices. In the present review, emphasis will be placed on some representative waste products.

### 2.1. Grape By-Products

Grape (*Vitis vinifera*) represents one of the most important horticultural crops in the world, with 78 million produced globally [15]. *Vitis vinifera* employed in the wine industry provides grapes, raisins, juices, and leaves as major by-products [16]. During grape vine production, leaves are discarded, but recently, their bioactive components, such as polyphenols and fibers, have been extracted and utilized [17]. Leaves contain six hydrobenzoic acids and six hydroxycinnamic acids, flavanols, and anthocyanins, which have been investigated in experimental studies [18].

There is evidence that caffeic and chlorogenic acids, and flavonoids, such as quercetin, contained in vine leaves, in vitro exert antioxidant activity, with the inhibition of linoleic acid peroxidation [19], while scavenging certain radicals, such as superoxide radicals and lipid peroxyl radicals [20]. In mice, quercetin protects the liver from oxidative stress, reducing the levels of nitric oxide (NO), while increasing the levels of glutathione peroxidase [20]. Also, ferulic acid has been shown to attenuate ethanol and carbon tetrachloride CO-induced hepatotoxicity in rats [21]. Evidence has been reported that aqueous grape leaf extract-mediated hepatoprotection depends on the activation of nuclear erythroid-related factor 2 (Nrf2), with the inhibition of glutathione reductase [22]. As far as neurodegeneration is concerned, in a model of Alzheimer’s disease, leaf polyphenols reduced neuronal damage caused by ROS, also attenuating neuroinflammation [23]. With special reference to adipogenesis, syringic acid promoted lipolysis in adipocytes via an ROS-mediated mechanism in high-fat diet (HFD)-induced obesity in mice [24]. Also, the antidiabetic activity of vine leaves has been ascribed to their content in condensed tannins, catechins, and procyanidins [25]. The anti-inflammatory activity of grape leaves depends on their ability to inhibit the translocation of Nf-kβ from the cytoplasm into the nucleus, with the decreased release of pro-inflammatory cytokines [i.e., IL-1, IL-6, and TNF-α] [26]. In fact, evidence has been provided that the highest dose of leaf extract reduced the levels of IL-6 by 60% and IL-8 by 40%, with IL-1β decreased to the basal level [27].

Grape pomace (GP) is a solid by-product from the wine-making process, enriched in skin and seeds, and 60–70% of polyphenols remain in the pomace after wine making [28]. GP is mainly composed of polyphenols and dietary fibers, which account for its properties [29]. GP polyphenols encompass hydroxybenzoic acids and hydroxycinnamic acids, stilbenes (resveratrol), and flavonoids [flavanols, anthocyanins (malvidin), and flavonols (quercetin, myricetin, and kaempferol)] [30].

Of note, dietary fibers are polysaccharides, such as cellulose, xyloglucans, arabinans, galactans, xylans, mannans, pectins, and lignin, which are bound to polyphenols, and other non-digestible compounds, the so-called “antioxidant dietary fiber” [31].

Then, the combination dietary fibers/polyphenols can enhance the healthy properties of grape by-products.

From an inflammatory and oxidative point of view, GP components have been shown to act on the NF-kβ and NrF2 pathway in rodents, pigs, and humans [32,33]. In addition, both polyphenols and dietary fibers, extracted from GP, suppressed the expression of TLR2 and TLR4 on intestinal cells [34,35,36]. In an experimental model of ulcerative colitis, GP led to a reduction in pro-inflammatory cytokines (IL-1, IL6, and TNF-α), intercellular adhesion molecule 1, and metalloproteinase 9, with an increase in the anti-inflammatory cytokine, IL-10 [37,38,39]. Noteworthy, polyphenols also exert anti-inflammatory activity inducing T regulatory cells, with the production of the anti-inflammatory cytokine, IL-10 [15,40]. In a similar experimental model, GP consumption resulted in the dramatic reduction in myeloperoxidase (MPO) activity, with the suppressed migration and infiltration of neutrophils into the intestinal mucosa [41].

Nrf2 is a pathway involved in antioxidant responses and sequestered in the cytoplasm by binding to a Kelch-like echt-associated protein 1 (Keap1) [42]. Keap 1 dissociates from Nrf2 when the concentration of ROS in the cytoplasm is upregulated, thus allowing Nrf2 to translocate to the nucleus and bind to the antioxidant response elements in its target genes. Therefore, such a process results in the generation of antioxidant enzymes, such as superoxide dismutase (SOD), catalase (CAT), and glutathione peroxidase (GPX) [43]. In this respect, the administration of GP to experimental models of inflammation led to an increase in SOD, CAT, and GPX, with reduced levels of ROS, NO, and iNOS, and enhanced activation of Nrf2 [44].

The effects of GP on the composition of the gut microbiota are under scrutiny. The gut microbiota can degrade GP components with the generation of flavonoids and short-chain fatty acids (SCFAs: butyrate, propionate, and acetate) from dietary fibers [45]. Then, these metabolites can modify the bacterial composition of the gut, either by reducing the formation of pathogen biofilms, e.g., *Escherichia coli*, *Actinobaceria*, and *Verrucomicrobia* or favoring the expansion of those bacteria, such as *Enterococcus*, *Prevotella, Bifidobacterium*, and *Faecalibacterium*, which can extract sugars from the complex polyphenols/dietary fibers for a probiotic effect to occur [46,47]. In large animals, GP administration promoted the proliferation of beneficial bacteria in the caecum and enhancement in antioxidant and anti-inflammatory activities, e.g., release of IL-10 [48,49]. Concerning human trials with grape by-products, the administration of Leucoselect Phytosome^®^ (a dietary supplement enriched in the flavonoid epigallocatechin) to frail elderly subjects was able to potentiate the otherwise impaired T helper 1 response [50]. Increased levels of IL-2 and interferon (IFN)-γ were observed, as an indication of the immune enhancement induced by polyphenols. In another trial, polyphenols extracted from the seeds of red grape (Nero di Troia cultivar) when administered to 25 patients affected by contact dermatitis to nickel could reduce the release of pro-inflammatory cytokines, while increasing the production of the anti-inflammatory cytokine, IL-10 [51]. In these patients, a parallel improvement in cutaneous lesions was also recorded. The ability of grape marc polyphenols to enhance the anti-inflammatory pathway has also been confirmed in vitro, using peripheral blood mononuclear cells (PBMCs) from healthy volunteers [52]. In fact, Negroamaro and Koshu *Vitis vinifera* extracts were able to in vitro expand Foxp3+ T regulatory cells, with increased production of IL-10. On the other hand, in subjects at risk of metabolic syndrome and cardiovascular disease, the increase in SCFAs and polyphenol metabolites after GP supplementation was not significant, with high interindividual variability related to differences in the gut microbiota and miRNA [53,54].

Conclusively, polyphenols contained in grape by-products are endowed with anti-inflammatory and antioxidant activities, and their daily intake, as in the case of a Mediterranean diet, may prevent the outcome of chronic disease. The effects of these compounds on the Gut microbiota with the protection of the intestinal barrier integrity should be taken into consideration. In this respect, a GP-mediated increase in *Lactobacillus duelddebruckii* enhances the intestinal barrier integrity in the gut with a higher expression of occludin and zonulin [45].

### 2.2. Olive By-Products

Olive pomace by-products consist of fruit pulp and husk, crushed olive stones, and wastewater. During olive oil extraction, 98% of the total phenolic compounds are present in pomace and wastewater, with oleuropein, hydroxytyrosol, tyrosol glucoside, and 4-hydroxyphenylacetic acid as major polyphenols [55,56].

Furthermore, pomace oil is enriched in oleic, palmitic, linoleic, and stearic acids [57]. Several studies on the effects of olive pomace have been conducted in vitro and in vivo. Using EAhy926 human endothelial cells under hypoxia, pomace extracts, enriched in hydroxytyrosol, tyrosol, and oleuropein, suppressed metalloproteinase inhibitor-1 and NF-kβ expression, as well as TNF-α, cyclooxygenase (COX)- 2, iNOS, and NO levels [58]. An ethanolic extract of oil pomace enriched in the above-mentioned polyphenols decreased the release of NO and NF-kβ in steatosis human endothelial cells [59], while in human corneal and conjunctival epithelial cells exposed to TNF-α and ultraviolet-B radiation, it suppressed the generation of pro-inflammatory cytokines and ROS [60]. In a model of dry eye disease, the same extract reduced the gene expression of cytokines (IL-1, IL-6, and TNF-α) and chemokines (IL-8 and monocyte chemoattractant protein-1 (MCP-1)) in the conjunctiva and lacrimal glands [61]. In HepG2 cells and nontumorigenic human hepatoma cells, pomace extracts prevented oxidative damage, and this activity was enhanced by cyclodextrin encapsulation in tert-butyl hydroperoxide-exposed cells [62]. In BV-2 microglial cells, activated by lipopolysaccharides, pomace reduced the generation of ROS and NO [63].

With special reference to clinical trials conducted with olive pomace, the administration of yogurt supplemented with olive pomace to healthy and overweight volunteers antagonized the action of platelet-aggregating factor (PAF), inhibiting PAF metabolism [64]. In healthy volunteers (aged 20–30 years) consuming 50 g of a functional biscuit, enriched with 20% olive paste, a decrease in serum triglycerides after 3 h was observed [65]. Furthermore, after 3 h, an increase in plasma antioxidant activity was recorded, likely due to the polyphenol and oleic acid contained in the biscuits.

Olive leaves represent another by-product accumulated by pruning olive trees and during oil extraction. Olive leaf extract (OLE) is highly enriched in polyphenols, such as tyrosol, hydroxytyrosol, oleuropein, catechin, caffeic acid, luteolin, and rutin, as major components [66]. Of note, there is evidence that the combination of various polyphenols exerts more beneficial effects than the individual one [67]. OLE possesses potent antioxidant activity with chemoprotective properties. In fact, hydroxytyrosol can mitigate the oxidative stress provoked by H_2_O_2_ and peroxynitrite on cells and DNA, thus blocking cell cycle advancement at the G1 phase, while inducing apoptosis [68].

Also, OLE oleuropein inhibits motility and cancer cell growth through apoptosis, thus reducing the incidence of cervical cancer cells, colon cancer, and breast cancer [69]. Quite interestingly, the removal of glucose from oleuropein decreases its inhibitory activity, thus suggesting a glucose-based entry pathway into the cell [70]. Therefore, cancer cells and overexpressing glucose transporters are more susceptible to oleuropein effects. In an in vitro study, PBMCs isolated from healthy volunteers, when treated with OLE extracts, underwent some changes, such as an increase in the number of natural killer cells and enhanced release of IFN-γ, thus indicating a potential reinforcement of the antitumor activity [71].

Evidence has been provided that oleuropein exerts cardioprotective effects, reducing ROS production and improving endothelial functions [72].

Furthermore, it lowers the blood vessel tension with more vessel spreading and antihypertensive effects [73]. Moreover, OLE polyphenols have been shown to prevent the formation of arterial plaques either by decreasing adhesion molecules on leukocytes and platelets or by preventing platelet aggregation [74]. Epidemiological and in vitro studies have demonstrated that OLE polyphenols can reduce the occurrence of age-related diseases. It has been reported that oleuropein could prevent or attenuate the aggregation of Ab peptides, which play a pathogenic role in the outcome of Alzheimer’s disease [75]. Moreover, polyphenol extracts play an anti-inflammatory effect that is very beneficial during aging-dependent pathologies, even including Alzheimer’s disease [76].

During the washing and processing of the oil fruit, the so-called oil mill wastewater (OMWW) is produced in large amounts [77]. OMWW is highly enriched in polyphenols, and, among them, hydroxytyrosol, tyrosol, oleuropein, verbascoside, vanillic acid, caffeic acid, p-coumaric acid, ferulic acid, and elenolic acid are the main components [78]. The antioxidant activity of OMWW has been investigated in vitro, using the AOO9 extract on HUVEC proliferation [79]. AOO9 inhibited ROS release before and after H_2_O_2_ treatment, as well as the in vitro migration and invasion of HUVEC [80]. Another OMWW extract, OliPhenolia^®^, performed a mild antioxidant effect on aerobic workout and acute recovery in healthy volunteers [81]. Experimentally, in old rats treated with OMWW, enriched in hydroxytyrosol, skeletal muscle decline due to age-related oxidative stress improved [82]. In terms of neuroprotection, in TgCRND8 mice, cognitive deficits and neuropathology were improved by OMWW extract, using a diet supplementation [83]. These in vivo data were supported by an in vitro study showing a decrease in the NO-induced cytotoxicity of murine brain cells following treatment with hydroxytyrosol-rich OMWW extract [84].

The anti-inflammatory effects of OMWW have been tested in in vitro experiments treating colorectal cancer cells with AOO9 [85]. In fact, AOO9 inhibited either the proliferation, migration, invasion, adhesion, and sprouting of cancer cells or release of pro-angiogenic and pro-inflammatory cytokines. Furthermore, OMWW exerted a cardioprotective effect during anticancer therapy with 5FU and cisplatin, reducing IL-6 mRNA [86,87].

### 2.3. Apple By-Products

Apples are the most consumed fruit, characterized by a high content of anthocyanins, hydroxycinnamic acid, quercetin, carotenoids, and vitamins C and E accounting for their antioxidant and anti-inflammatory activities [88]. The use of apples and their by-products (pomace and peel) is very common in the preservation of food products, as in the case of fish protection from oxidation [89]. Moreover, apple polyphenols from pomace and peel contribute to retaining the quality of meat and meat products [90,91].

Quite interestingly, the incorporation of apple pomace into noodles, vegetable juices, and yogurt increased the nutritional properties of these food products in terms of dietary fibers and protein content, as well as the potentiation of antioxidant activity [92,93]. In particular, the presence of polyphenols may account for their anti-inflammatory and antioxidant activities, with a reduction in pro-inflammatory cytokines (IL-1, IL-6, and TNF-α) and increase in the release of the anti-inflammatory cytokine, IL-10. At the same time, one cannot exclude the effects of apple by-products on the intestinal microbiota with an increase in SCFAs. In this respect, in weaning piglets fed apple by-products, modifications of the intestinal architecture and function, as well as modulation of the intestinal microbiota, have been documented, along with the production of lactobacilli and *Faecalicaterium* spp. [94].

These data suggest the potential application of apple by-products to human studies. However, with special reference to safety issues, apple by-products contain pesticide residues, and apple seeds can release cyanide glycosides, thus suggesting the need to pay caution before use in humans [95].

### 2.4. Pumpkin By-Products

Pumpkin is a vegetable highly enriched in healthy bioactive substances, such as polyphenols (curcumin, flavonoids, lignans), carotenoids, tocopherols, fatty acids, vitamins C and E, linoleic acid, and selenium [96]. Pumpkins and different parts of pumpkins can be used as functional ingredients for industrial applications. Pumpkin seed oil is a by-product enriched in carotenoid pigments, with the ability to reduce the risk of colon and lung cancer [97]. Seed oil could be protective during subacute aflatoxin poisoning in mice, in view of its antioxidant properties [98].

In rats with nonalcoholic fatty liver disease treated with biscuits containing 15% pumpkin seed meal and 3% seed oil, a reduction in lipidemia and transaminase activity was observed [99].

Moreover, pumpkin seed intake is associated with a decreased risk of Alzheimer’s disease, while lowering blood sugar in animals with impaired glucose metabolism [100,101]. The hypoglycemic effect of pumpkin seems to depend on polysaccharides, as observed in alloxan-mediated diabetic rats [102]. Also, seed proteins and pumpkin leaves were effective in preventing protein-energy malnutrition-induced oxidative brain damage in rats [103]. As far as the anti-inflammatory activity of pumpkin is concerned, pumpkin seed oil attenuated adjuvant-induced arthritis in rats, reducing free-radical release [104]. There is evidence that in mice, the antioxidant activity exerted by pumpkin fruit extracts depends on the increased function of glutathione peroxidase and SOD with a reduction in the malonaldehyde concentration [105].

Quite interestingly, pumpkin skin is fermented by human fecal microbiota, thus promoting SCFA production, with the promotion of a healthy microbiota rich in *Lactobacillaceae* and *Faecalibacterium* [106].

### 2.5. Tomato By-Products

The tomato processing industry is one of the largest in the horticultural area, characterized by enormous waste and by-product accumulation. Lycopene, a carotenoid, is the main compound in by-products and its valorization is under investigation, in view of high contents in polyphenols (rutin, quercetin, caffeic acid, and kaempferol), dietary fibers (polysaccharides, oligosaccharides, and lignin), oil, protein, and organic acids [107].

By-products encompass peels, seeds, pulp, rotten tomatoes, green tomatoes, and tomato branches.

Lycopene possesses antioxidant properties, scavenging free radicals, and neutralizing singlet oxygen and sulfonyl radicals [108]. The antioxidant effect of lycopene can be enhanced by carotenoids and vitamins (vitamins E and C), mostly scavenging reactive nitrogen and lipid peroxidation [109].

Lycopene has been shown to in vitro inhibit ROS generation, preventing low-density lipoprotein oxidation [110]. Furthermore, it can prevent endothelial injury, modulate lipid metabolism, and reduce the levels of IL-1, IL-6, and TNF-α as well as platelet aggregation [111].

The above effects indicate the potential cardioprotective activity of lycopene. In experimental animals, lycopene administration was able to reduce pro-inflammatory cytokine and transforming growth factor-β (TGF-B) release in the brain [112]. In eye disorders, e.g., non-proliferative diabetic retinopathy, carotenoid supplementation could increase the optical density level of the macular pigment, thus suggesting the protective effects of tomato by-products in various eye disorders.

Experimentally, tomato powder and distilled lycopene exert anti-neoplastic activities, as in the case of prostate cancer and MCF7 human mammalian cancer cells [113].

In general terms, lycopene modulates immune responses, inhibiting iNOS, COX-2, and lipoxygenase expression, as well as NF-kβ activity [114]. In addition, lycopene activates the T cell-dependent adaptive immune response of mostly T helper 17 cells, with the production of IL-17, thus protecting against bacterial infection [115].

Of note, quercetin is a polyphenol, contained in tomato by-products, which enhances the beneficial effects exerted by lycopene [116].

It is conceivable that tomato by-products may modulate the gut microbiota with the same mechanisms illustrated in the previous sections.

### 2.6. Rice By-Products

Rice is one of the most produced cereals in the world, providing a great caloric contribution to the human diet. It contains carbohydrates (80%), proteins (7–8%), fats (3%), minerals (6–7%), and fibers (3%), as well as tocopherol and tocotrienols [117].

The kernel represents 70% of the rice grain, while rice by-products consist of husk, bran, and germ [118]. Rice bran (the outer layer of the rice grain) is added to bread, thus increasing the content of vitamin B, fibers, and minerals [119]. Rice bran oil is enriched in ferulic, gallic, coumaric acids, cyanidins, gamma-oryzanol, and vitamin E with anti-inflammatory and antioxidant activities but use in humans is limited because of its high levels of free fatty acids [120]. Rice germ is richer in vitamin E than rice bran and contains essential amino acids, linoleic, and linolenic acids, thus suggesting its potential application to humans [121]. Furthermore, evidence has been provided that the enzymatic degradation of dietary fibers in rice grains generates oligosaccharides with prebiotic effects at the intestinal level [122].

Conclusively, rice by-products and, especially, rice bran have the potential for industrial applications, such as fortified foods and/or supplements, endowed with health-promoting properties; however, more studies are needed for their better exploitation. In Table 1, the components and bioactivity of plant by-products are summarized.

**Figure 1:**
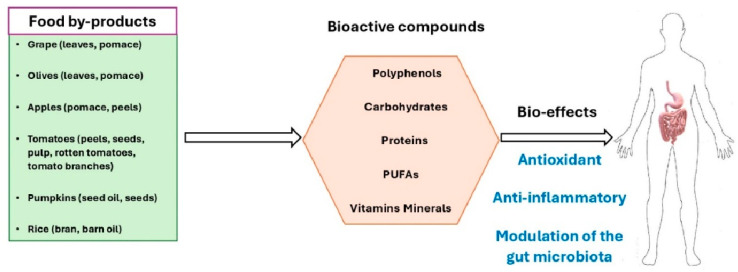
Principal by-products from fruit, vegetables, and cereals. Food by-products are endowed with three main effects, namely, antioxidant, anti-inflammatory, and gut microbiota modulatory activities.

By-products from fruit, vegetables and cereals, with their specific bioactive compounds and related biological effects, are summarized in Figure 1.

## 3. By-Products from Marine Food Sources

Among marine foods, microalgae exert beneficial effects when ingested with diet. For this reason, food industries have increased the production of food enriched in microalgae, growing them in open ponds and photobioreactors [123]. Different bioactive molecules can be isolated from microalgae, especially diatoms (*Bacillariophyceae*), such as carotenoids, polysaccharides, polyphenols, sterols, proteins/peptides, and fatty acids [124].

Fucoxantin, a carotenoid extracted from brown microalgae, in vitro exerts antioxidant and anti-inflammatory activity on BV-2 microglial cells, reducing the release of IL-1, IL-6, and TNF-α [125].

Polyphenols are secondary metabolites of microalgae endowed with antioxidant activities [126]. Experimentally, *Nitrshia (N.) palea* polyphenols, using Raw 264.7 macrophages, inhibited the expression of pro-inflammatory cytokines and NO [127].

Oral administration of *N. palea* to mice with carrageenan-induced edema reduced edema via the local inhibition of COX-2 and MPO activity [127].

With special reference to PUFAs, *Pinnularia borealis* accumulates high amounts of eicosapenteaenoic acid (EPA), and its supplementation to mice improved antioxidant defenses and risk of cardiovascular disease [128]. Other studies have revealed the ability of fatty acids extracted from *Anomoenois* sp. and *Rhoipolodia* sp. to inhibit angiotensin-converting enzyme, thus affording cardioprotection to the host [129]. In 19 healthy elderly subjects, the administration of EPA extracted from *Phaeodactylum (P.) tricornutum* generated anti-inflammatory and antioxidant effects.

Sterols, a class of triterpenoid molecules extracted from microalgae, possess antioxidant, anti-inflammatory, anticancer, and anti-cholesteroligenic activities [130].

Brassicasterol prevented atherosclerosis and induced hypocholisterolemia; campesterol was effective in cancer models; fucosterol and isofucosterol were protective in diabetes models; and sistosterol was able to lower cholesterol [131].

Polysaccharides are generated in the chloroplast and cytosol of microalgae, where they are present together with proteins and long-chain polyamines [132].

Leucosin or chrysolaminarin, isolated from *Ondotella aurita* and *P. tricornutum,* showed antioxidant activity in vivo, using flat fish as a model [133].

Furthermore, a hydrosoluble extract of *P. tricornutum* reduced carrageenan-induced paw edema and delayed hypersensitivity in rats, potentiating in vitro phagocytosis [134]. The effects of polysaccharides from the microalgae Spirulina (*Arthrospira platensis*) have been explored [135]. Spirulina polysaccharides administered to normal mice could increase phagocytosis and pro-inflammatory cytokine release via the activation of the NF-kβ, MAPK, and JAK/STAT pathways [136]. In cyclophosphamide-immunosuppressed mice, Spirulina polysaccharides improved the function of monocytes and B lymphocytes [137].

In zebra fish, polysaccharides extracted from Spirulina maximally stimulated the innate immune response against *Aeromonas hydrophila* and *Edwardsiella piscicida* [138].

As far as antioxidant activity is concerned, Spirulina polysaccharides inhibit lipid peroxidation, while increasing the SOD and CAT activities [139,140]. In HFD mice, Spirulina polysaccharide administration increased the number of beneficial bacteria (*Bacteroidetes* and *Mollicutes*), while decreasing the number of dangerous bacteria (*Actinobacteria* and *Verrucomicrobia*), as well as reducing the risk of obesity [141]. Moreover, in mice, Spirulina polysaccharides improved constipation symptoms, as well as liver inflammation [142,143].

Proteins from *N. laevis* have been demonstrated to convert angiotensin-I to angiotensin II, playing both antihypertensive and antioxidant roles, as well as proteins from *Bellerochea mallus* dramatically reducing blood pressure in spontaneous hypertensive rats [144].

Arthropods (e.g., crabs, lobsters, and shrimp) are rich sources of chitin with chitooligosaccharides (COS) as major by-products [145]. COS derived from chitin and chitosan by acid or enzymatic hydrolysis have been shown to exhibit antioxidant, anti-inflammatory, immune-enhancing, and antitumor activities [146]. In this respect, COS reduced the production of NO, iNOS, and prostaglandin E2 in Raw 264.7 macrophages [147]. Moreover, they exerted a protective effect against H_2_O_2_-induced umbilical vein endothelial cell apoptosis and oxidative damage [148].

Quite interestingly, low-molecular-weight COS have been demonstrated to exert the most potent anti-inflammatory activity, with a reduction in NO, iNOS, and COX-2 expression [149].

In light of the above results, it has been suggested to employ metabolic engineering techniques to produce tailor-made COS products to be applied to human studies [150].

Of note, large amounts of waste of fish scales, skin, and bones have attracted attention. For instance, collagen peptides isolated from milkfish scales have been shown to possess antioxidant and anti-inflammatory activities, as well as DNA-protective activities [151]. Therefore, these products may have potential application in cosmeceuticals and in nutraceuticals. The main characteristics of marine food by-products are illustrated in Table 2.

## 4. Effects of Food By-Products on the Intestinal Microbiota

The normal microbiota is characterized by a high diversity with a predominance of *Firmicutes* and *Bacteroidota* for maintining a healthy gut environment [47]. Dysbiosis depends on the disruption of the intestinal microbiota composition, thus leading to disease, such as inflammatory bowel disease [46].

Dietary fibers are major by-products extracted from vegetables and fruit, as well as seaweeds, and largely utilized by the food industry to promote the circular economy [152]. Dietary fibers are non-starch polysaccharides neither digested nor absorbed in the human intestine, including pectin, inulin, chitins, and β-glucans [153]. Pectin is a complex of acidic heteropolysaccharides based on homogalacturonan, rhamnogalacturonan I (RGI) and RGII, and xylogalacturonan [154]. Inulin is mainly made up of β-(2-1)-fructosyl-fructose linkages [155]. Alginate is a polysaccharide contained in the cell wall of brown seaweeds, and composed of two hexuronic acids, D-mannuronic acid and L-guluronic acid [156]. The above-mentioned dietary fibers are fermented by the colonic microbiota, exerting prebiotic and antioxidant effects in the host [157,158].

In a model of obese colitis mice, pectin administration increased the ratio between *Bacteroidetes* and *Firmicutes*, reducing the secretion of IL-1β and IL-6, with the attenuation of colitis [159]. Further evidence has been provided that the coadministration of pectin and polyphenols was very effective in the modulation of the gut microbiota in obese mice or mice with colitis via the inhibition of the NF-kβ pathway and cytokine secretion [160,161,162]. Moreover, dietary fiber administration to obese animals prevented metabolic abnormalities, with SCFA production and increased expression of uncoupling protein 2 ultimately stimulating the oxidative metabolism in the liver and adipose tissue via AMPK [163].

Alginate administration to mice reduced *Salmonella* and *Staphylococcus* inflammatory capacity [164]. In obese mice, the administration of alginate increased the ratio of *Bacteroidetes/Firmicutes* and the levels of *Bifidobacteria* and *Prevotella,* with the production of succinate and lactate, thus promoting healthy benefits, such as improvement in glucose homeostasis and attenuation of obesity-associated disorders [165].

Inulin has been used in various clinical trials. In patients with chronic kidney disease, inulin administration increased the number of *Bifidobacterium* and *Lactobacillaceae* with the production of SCFAs and decrease in C-Reactive Protein, TNF-α, and NADPH Oxide-2 [166]. In obese women, inulin administration increased the number of beneficial bacteria, such as *Faecalibacterium (F.) prausnitzii*, with an enhanced production of SCFAs [167]. In another group of obese individuals, the coadministration of inulin and polyphenols increased the production of SCFAs [168]. In the same direction, the combination of inulin and pectin was beneficial in patients with type 2 diabetes (T2D), and in obese patients through increased levels of *F. prausnitzii*, decreased levels of *Roseburia*, and generation of anti-inflammatory activity, as well as better sensitivity of insulin for T2D patients [169].

Conclusively, dietary fiber by-product administration generates a remarkable amount of SCFAs in the gut, thus correcting microbiota alterations.

**Figure 2:**
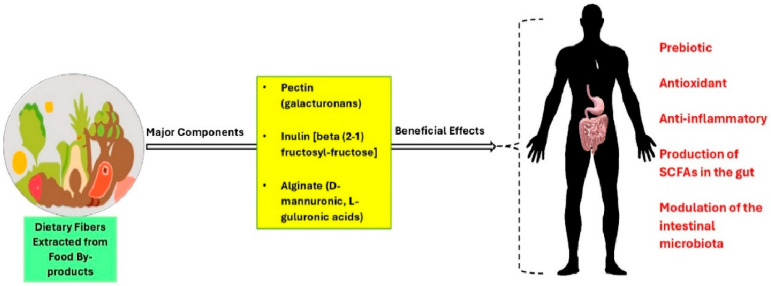
Major components of dietary fibers. Fermentation of dietary fibers in the gut leads to beneficial effects in the host, even including modulation of the intestinal microbiota.

Major features of dietary fiber by-products are shown in Figure 2.

## 5. Conclusions

Over recent years, the human use of natural products has been rapidly growing. Fruit, vegetables, cereals, and or their derivatives (e.g., wine and olive oil) have been shown to contribute to human health, as in the case of a Mediterranean diet. Food and marine food by-products represent another enormous source of bioactive compounds, and, among them, polyphenols, proteins, polysaccharides, and dietary fibers are the most exploitable ones for preventive and therapeutic use. At the same time, the use of by-products avoids their indiscriminate accumulation in the environment, supporting the concept of the circular economy.

Some by-products are not fully exploited and related research is limited to in vitro and in vivo models. Furthermore, their formulation as encapsuled agents is under scrutiny. Moreover, modulation of the gut microbiota represents another beneficial property of food by-products with the enhancement in the intestinal barrier and decreased dissemination of intestinal pathogens to systemic circulation. Considering the increase in chronic disease and cancer, mostly associated with obesity and T2D, dietary by-products may represent future sources of bioactive products for human use. Finally, persistent consumption of vegetable and marine by-products usually also has the advantage of being free of side effects.

## Figures and Tables

**Table 1:** A general view of plant by-products.

GRAPE BY-PRODUCTS
Major components: polyphenols, fibers [17].
Activities
In vitro anti-inflammatory activities with activation of T regulatory cells and release of the anti- inflammatory cytokine, IL-10 [15,32,33].
In obese rats, modulation of gut microbiota with production of SCFAs and expansion of *Enterococcus*, *Prevotella*, *Bifidobacterium*, and *Faecalibacterium* [45].
Potentiation of T helper 1 activity in elderly subjects [50], and mitigation of symptoms in contact dermatitis to nickel in patients, with production of IL-10 [51,52].
OLIVE BY-PRODUCTS (olive pomace, olive leaf extract, and oil mill wastewater)
Major components: polyphenols, fatty acids, stearic acids, carotenoids, vitamin C and E [55,56,57,96].
Activities
In animal models, anti-inflammatory activities (inhibition of NF-kβ, TNF-α, PAF), increase in antioxidant activities, and decrease in serum triglycerides [64,65,68,69,79,81].
In animal models, cardioprotective effects and prevention of arterial plaque formation [70,74].
In vitro, inhibition of proliferation and angiogenesis in cancer cells [85].
In animal models, prevention of Alzheimer’ disease outcome with attenuation of Ab peptide aggregation [75].
In obese rats, effects on the gut microbiota with increase in SCFAs and enhancement in intestinal barrier integrity [87].
APPLE BY-PRODUCTS
Major components: polyphenols, carotenoids, vitamins C and E.
Activities
Antioxidant and anti-inflammatory activities [88].
In piglets, effects on the gut microbiota with expansion of lactobacilli and *Faecalibacterium* [94].
PUMPKIN BY-PRODUCTS
Major components: polyphenols, carotenoids, vitamins C and E, linoleic acid, selenium [96].
Activities
In animal models:
Decreased risk of Alzheimer’s disease [100,101].
Prevention of energy malnutrition [103].
Attenuation of arthritis [104].
Increase in SCFA production in the gut and expansion of *Lactobacillaceae* and *Faecalibacterium* spp. [106].
TOMATO BY-PRODUCTS
Major components: polyphenols, dietary fibers, proteins, lycopene [107].
Activities
In animal models:
Lycopene-mediated anti-inflammatory and cardioprotective effects [111,112].
Lycopene-mediated production of IL-17 with protection against bacterial infections [115].
RICE BY-PRODUCTS
Major components: carbohydrates, proteins, fats, minerals, fibers, polyphenols, vitamin E [117].
Activities
In animal models:
Enzymatic degradation of dietary fibers followed by generation of oligosaccharides in the gut with probiotic effects [122].
In humans:
Limited use of rice bran oil for its high levels of free fatty acids [120].

**Table 2:** General view of marine by-products.

MARINE-BY PRODUCTS
MICROALGAE Major components: carotenoids, polysaccharides, polyphenols, sterols, proteins/peptides, and fatty acids [124]. Activities Fucoxantin (carotenoid): in vitro antioxidant and anti-inflammatory activities [125]. Polyphenols: in vitro inhibition of pro-inflamamtory cytokine and nitric oxide [127]. Polyunsaturared fatty acids: in animal models, antioxidant, anti-inflammatory, and anti-cholesteroligenic activities [130]. Polysaccharides (Spirulina): in animal models, increase in innate immunity functions and B cell activities [135,136,137,138], inhibition of lipid peroxidation [139,140], modulation of gut microbiota [141], and improvement in constipation symptoms and liver inflammation [142,143]. Proteins: in animal models, antihypertensive and antioxidant roles [144].
ARTHROPODS Major component: chitooligosaccharides (COS) [145]. Activities COS: in vitro, antioxidant, anti-inflammatory, immune-enhancing, and antitumor activities [145,146,147,148,149].
FISH BY-PRODUCTS (fish scales, skin, bones) Activities Collagen peptides from fish scales: in vitro antioxidant, anti-inflammatory, and DNA-protective functions [151].
